# Glucosinolate and Sugar Profiles in Space-Grown Radish

**DOI:** 10.3390/plants14132063

**Published:** 2025-07-06

**Authors:** Karl H. Hasenstein, Syed G. A. Moinuddin, Anna Berim, Laurence B. Davin, Norman G. Lewis

**Affiliations:** 1 Biology Department, University of Louisiana Lafayette, Lafayette, LA 70504-3602, USA; 2 Institute of Biological Chemistry, Washington State University, Pullman, WA 99164-7411, USA; syed@wsu.edu (S.G.A.M.); aberim@wsu.edu (A.B.); davin@wsu.edu (L.B.D.); lewisn@wsu.edu (N.G.L.)

**Keywords:** Brassicaceae, *Raphanus sativus*, carbon dioxide, glucosinolates, sugars, microgravity, space cultivation, International Space Station, Advanced Plant Habitat, HPLC, UPLC-qTOF-MS

## Abstract

The quest to establish permanent outposts in space, the Moon, and Mars requires growing plants for nutrition, water purification, and carbon/nutrient recycling, as well as the psychological well-being of crews and personnel on extra-terrestrial platforms/outposts. To achieve these essential goals, the safety, quality, and sustainability of plant material grown in space should be comparable to Earth-grown crops. In this study, radish plants were grown at 2500 ppm CO_2_ in two successive grow-outs on the International Space Station and at similar CO_2_ partial pressure at the Kennedy Space Center. An additional control experiment was performed at the University of Louisiana Lafayette laboratory, at ambient CO_2_. Subsequent analyses of glucosinolate and sugar species and content showed that regardless of growth condition, glucoraphasatin, glucoraphenin, glucoerucin, glucobrassicin, 4-hydroxyglucobrassicin, 4-methoxyglucobrassicin, and three aliphatic GSLs tentatively assigned to 3-methylpentyl GSL, 4-methylpentyl GSL, and *n*-hexyl GSL were present in all examined plants. The most common sugars were fructose, glucose, and sucrose, but some plants also contained galactose, maltose, rhamnose, and trehalose. The variability of individual secondary metabolite abundances was not related to gravity conditions but appeared more sensitive to CO_2_ concentration. No indication was found that radish cultivation in space resulted in stress(es) that increased glucosinolate secondary metabolism. Flavor and nutrient components in space-grown plants were comparable to cultivation on Earth.

## 1. Introduction

The identification and characterization of plants is an ancient discipline, and modern analytical techniques have discovered an ever-increasing array of phytochemicals and metabolic pathways. This work has resulted in the identification and quantification of a myriad of compounds often associated with human health benefits. Such secondary metabolites, i.e., those that are not part of metabolites linked to energy formation or conversion, are as diverse as the plant kingdom and often specific for a subset of species within a phylogenetic group. Glucosinolates (GSLs) are characteristic of the plant order Brassicales, which includes the model plant Arabidopsis [1]. Many of the edible plants in this order are valued for their health benefits, but sometimes disliked because of their pungent flavor, such as cabbage (*Brassica oleracea*), broccoli (*Brassica oleracea* var. *italica*), brussels sprouts (*Brassica oleracea* var. *gemmifera*), horseradish (*Armoracia rusticana*), wasabi (*Wasabia japonica*), and radishes (*Raphanus sativus*). The often-cited bland flavor of space food [2] and the numerous health benefits of GSLs make Brassicales-based vegetables an important space crop food that are likely to provide spice to astronaut food, psychological benefits, gustatory appreciation, and also benefit the general public.

GSLs contain sulfur, nitrogen, and glucose moieties. A specific enzyme, myrosinase, hydrolyzes GSLs into isothiocyanates, which are responsible for the characteristic flavor [3,4], antimicrobial activity [5], and health benefits [6,7]. GSL formation is sensitive to light [8,9], UV light [10], temperature [11], herbivory [12], and soil properties [13]. GSLs are biosynthesized from the amino acids methionine, phenylalanine, and tryptophan, resulting in GSLs that can be termed aliphatic, aromatic, and indolic, respectively. Vegetables can also contain aliphatic GSLs derived from other amino acids, such as the branched-chain amino acids valine, leucine, and isoleucine [14,15,16,17].

Because of their complex metabolic activities, flavor profiles, and generic health benefits, it stands to reason that cruciferous vegetables will be beneficial to astronauts and the development of future outposts on the Moon and Mars. Although hyper-gravity appears to lower GSL concentrations [18], the effect of reduced gravity on glucosinolate levels has not been investigated. While some indications exist that plants respond to the entire gravity spectrum from micro- to hyper-gravity [19], the effect of space cultivation on plants is more complex than just altering the magnitude of the gravity vector. Secondary effects, such as increased CO_2_, elevated levels of volatile organic compounds (VOCs), and altered (gas) buoyancy, also affect plant growth.

Because space flight affects sugar composition and quantity [20], and no characterization of sugars exists for space-grown radishes, we compared the GSL and monosaccharide/disaccharide spectrum of radish plants grown in the Advanced Plant Habitat (APH) on the International Space Station (ISS) to ground controls [21]. Despite consistent biomass yields in space and Earth-grown plants [22], both glucosinolates and sugars varied widely in quantity and composition.

## 2. Results

### 2.1. Glucosinolates

GSLs have been extensively studied in radish root tissues, with glucoraphasatin being the most abundant; other less abundant GSLs include glucobrassicin, 4-hydroxyglucobrassicin, 4-methoxyglucobrassicin, glucoraphenin, glucoerucin, and aliphatic GSLs (tentatively annotated as 3-methylpentyl GSL, 4-methylpentyl GSL, and *n*-hexyl GSL) [23,24,25,26,27,28]. In this investigation, the GSLs from ISS grown radish bulbs and the corresponding ground controls were identified by retention time (R_t_), observed and calculated accurate mass spectroscopic molecular ions, selected molecular ion monitoring (SIM) [M–H]^–^, and fragmentation ions [25,29,30], in addition to the use of glucoraphasatin as an authentic standard (Table 1 and Supplemental Figure S1).

**Figure 1:**
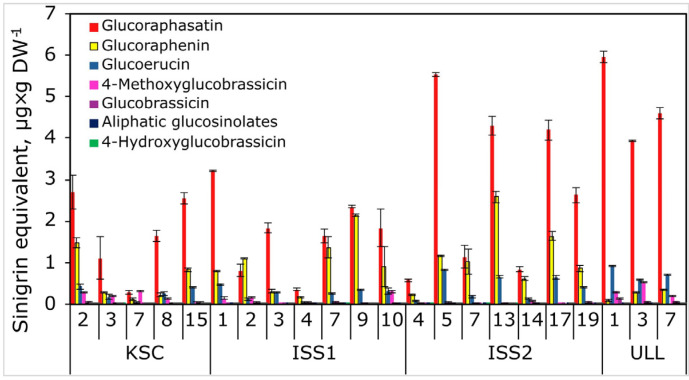
Relative quantities of glucosinolates extracted from radish bulbs grown under 2500 ppm CO_2_ (KSC, ISS1, and ISS2) and ambient CO_2_ levels (ULL) with individual replicate samples. The numbers refer to individual plant seed designations in the Science Carrier of the Advanced Plant Habitat (APH). Data are shown as sinigrin equivalents based on its use as an internal standard (IS) added to each sample. Aliphatic glucosinolate values are the total of provisionally annotated 3-methylpentyl GSL, 4-methylpentyl GSL, and *n*-hexyl GSL, respectively.

The distribution of GSLs in all samples shows most prominently glucoraphasatin and glucoraphenin, followed by glucoerucin and 4-methoxyglucobrassicin (Figure 1 and Supplemental Figure S2 for chemical structures of radish glucosinolates).

Glucobrassicin, 4-hydroxyglucobrassicin, and tentatively annotated aliphatic glucosinolates 3-methylpentyl GSL, 4-methylpentyl GSL, and hexyl GSL combined (Figure 1) were least abundant under high CO_2_ conditions (KSC and ISS grow-outs) but were generally more abundant in the university laboratory (ULL) samples at ambient CO_2_. By contrast, glucoerucin and 4-methoxyglucobrassicin were more abundant than glucoraphenin in the ULL samples. Despite these general observations, the data clearly showed that even within the same sample group, the quantity of GSLs varied considerably. The ratio between glucoraphasatin and the next most abundant compound, glucoraphanin, varied between 67:1 (ULL) and 0.7:1 (ISS1-2). These results indicate (1) no consistent relationship between the GSL profile in space and ground controls, and importantly, (2) the space experiments showed similar variability in the GSL profiles as the ground controls (Figure 1).

The tentatively annotated aliphatic GSLs were initially based on *n*-hexyl isothiocyanate synthesis [31]. This standard was later used for verification of *n*-hexyl GSL, based on the presence of its *n*-hexyl isothiocyanate derivative in steam distillates of fresh Japanese radishes “Miura Daikon” [32], where 4-methylpentyl isothiocyanate, the presumed derivative of 4-methylpentyl GSL, was also detected. By contrast, 3-methylpentyl GSL was later isolated and fully characterized from *Cardamine diphylla* [33]. The 4-methylpentyl isothiocyanate derivative was also reported in radishes [34], this perhaps only being based on the mass spectra obtained and not by comparison with an authentic standard.

All three tentatively annotated aliphatic GSLs were detected in our investigation and have been suggested to be present in numerous Brassica vegetables, including radish [25,30], but not confirmed with authentic standards. The order of elution (UPLC CSH C18 column) is indicated in Figure S1H and corresponds to previous reports [25,26,27,28,35,36]. All three were detected in this investigation, which is consistent with Kjaer et al. [32], reporting 4-methylpentyl- and *n*-hexyl isothiocyanates but not the 3-methylpentyl thiocyanate derivative.

**Figure 2:**
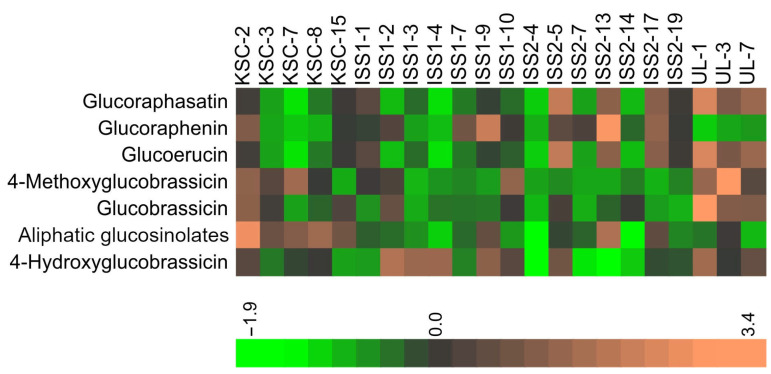
Heatmap of radish bulb glucosinolates. Data were normalized based on the average and STD of each glucosinolate for all observations (KSC, ISS1, ISS2, and ULL). Aliphatic glucosinolate composition is detailed in Figure 1.

The highly variable quantities of individual GSLs in samples across the examined conditions resulted in no readily discernible pattern in the heatmap of the GSLs (Figure 2).

**Figure 3:**
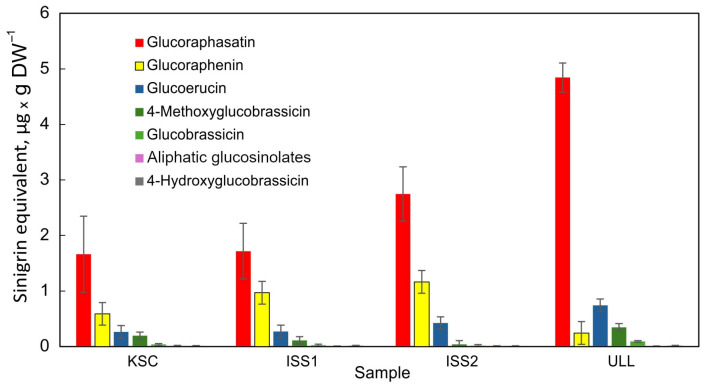
Averaged distribution of GSLs in radish bulbs across all measured samples from each experimental group. Error bars are based on the geometric mean of samples in each group. Tentatively annotated aliphatic GSL composition is detailed in Figure 1.

However, averaging data within a group showed a more consistent pattern of relative distributions of GSLs from the various grow-outs (Figure 3); that is, the average of replicates yielded a more consistent GSL profile for KSC, ISS1, and ISS2 with an exponential decline in the GSLs from the highest (glucoraphasatin) to the less abundant GSLs, glucobrassicin, 3-methylpentyl GSL, 4-methylpentyl GSL, *n*-hexyl GSL, and 4-hydroxyglucobrassicin. There was also no statistically significant difference in glucoraphasatin levels between the KSC and either space grow-out. In contrast, the ambient CO_2_ cultivation condition in the ULL laboratory showed significantly increased glucoraphasatin and diminished glucoraphenin levels, respectively.

Plants grown under high CO_2_ (KSC, ISS1, and ISS2) had lower glucoraphasatin content than ULL-grown plants (*p* = 0.003, df = 22). Additionally, glucoerucin was the second most abundant GSL under ambient CO_2_ (ULL), in contrast to all other experimental groups, and was significantly higher than in the high CO_2_ condition samples (*p* = 0.002, df = 22). There was no difference between high and low CO_2_ conditions for glucoraphenin and all other GSLs. Similarly, there was no significant difference between the quantity of GSLs in space-grown and Earth-grown plants.

### 2.2. Sugar Analyses

**Figure 4:**
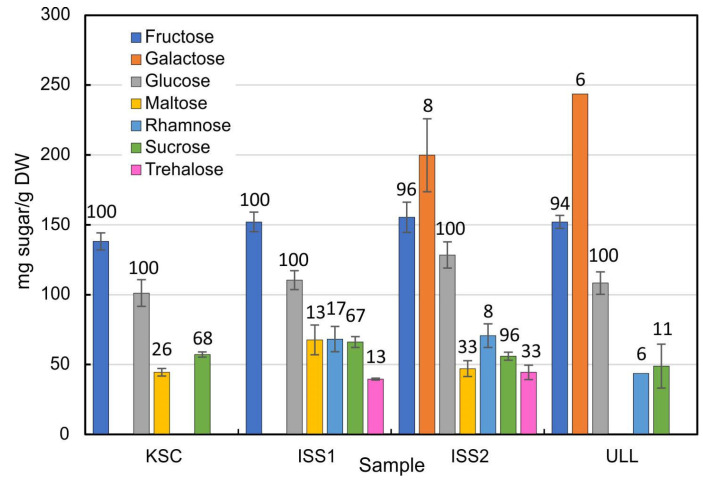
Quantities of sugars extracted from radish bulbs grown under 2500 ppm CO_2_ (KSC, ISS1, and ISS2) and ambient CO_2_ (ULL). KSC and ULL plants were grown at 1*g*; ISS samples were grown in weightless conditions (microgravity) in the APH on the International Space Station. The numbers above the bars represent the percentage of samples that contained the reported sugar.

Because glucose is a common sugar entity in GSLs, and together with other carbohydrates, a critical component of general metabolism, we investigated the distribution of common mono- and disaccharides in radish bulbs (Table 2; Figure 4).

The distribution of carbohydrates in radish plants showed fairly consistent quantities for fructose, glucose, and sucrose. Space-grown plants exhibited the most diverse spectrum of sugars, as ISS1 and ISS2 samples contained six and seven individual sugars, respectively (Figure 4). None of the 1*g* (KSC and ULL) samples apparently contained trehalose. Surprisingly, galactose was the dominant sugar in ISS2 and in the ULL-grown plants but was not detected in KSC and ISS1 plants. Maltose was detected in KSC, ISS1, and ISS2 plants but not in ULL samples. Additionally, rhamnose was only present in 6–8% of the ISS2 and ULL bulb specimens.

**Figure 5:**
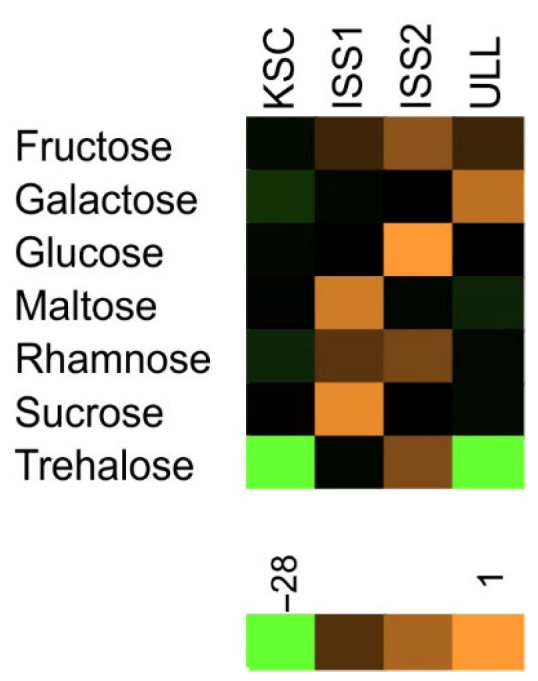
Heatmap of sugars contained in the bulb tissue of *Raphanus sativus*. Data were combined for all observations (KSC, ISS1, ISS2, and ULL) and normalized based on the average and STD of each sugar.

As for the prevalence of sugars in individual samples (percentages in Figure 4), it is important to point out that in addition to quantitative differences of sugars, there was no consistent representation between samples from the same experimental group. While fructose and glucose were present in most samples, sucrose was measured in more than half of all samples with elevated CO_2_. Conversely, plants grown in ambient CO_2_ (ULL) were less likely to contain sucrose in appreciable quantities and lacked maltose. In contrast to the illustration of glucosinolates, a heatmap of radish sugars (Figure 5) identifies the elevated concentrations in space-grown plants and the absence of trehalose in ground controls. Although the presence of trehalose was not uniform in all space-grown samples, the observation that only space-grown samples contained this disaccharide suggests a shift in sugar metabolism that is linked to space-grown plants.

Both the difference in quantity and occurrence in individual samples highlight the enormous metabolic diversity of radish sugars under varying environmental conditions.

## 3. Discussion

*Glucosinolates:* The observed variety and quantity of major GSLs corresponds to previous reports of radish metabolites [25,26,27,28,35,36], with each GSL in this study being detected via their HRMS accurate mass molecular ions [M–H]^–^ and ion fragmentation patterns and compared with previous reports (Table 1 and Supplemental Figure S1 as an example). Although glucoraphasatin was the most dominant GSL in all samples, the content varied greatly between and among the different culture conditions, as was also reported previously [25]. Requirements for GSL biosynthesis, however, depend on many factors, from glucose to sulfur, nitrogen, the complex networks from amino acids linking to transcription factors, and plant hormones such as jasmonic acid and auxin, as well as mineral nutrition [37,38]. Taken together, this suggests that GSL biosynthesis is capable of adapting to—or being regulated by—many factors. While the variability in quantity for individual GSLs showed no differentiation between space- or Earth-grown plants, the data suggest that GSL biosynthesis was responsive to the partial pressure of CO_2_. Other studies suggest that larger metabolic effects of elevated CO_2_ levels occur at about 700 ppm [39], considerably less than the 2500 ppm used in our experiment.

*Carbohydrates:* Although sugars were analyzed in space-grown wheat (*Triticum aestivum* L.) [40], there are no reports on the effect of weightlessness on low-molecular-weight carbohydrates. Our results (Figure 4) indicate a consistent representation of fructose, glucose, and sucrose, but the remainder of the measured sugars showed substantial variability. These observations are in line with the reported insensitivity of bulk sugar metabolism in hyper-gravity experiments [41] and the average sugar contents of 82 radish accessions [42], but inconsistent with the increase in foliar carbohydrate levels in response to hypoxia [43]. Although elevated CO_2_ was associated with an increased hexose to sucrose ratio [44], this effect was short-lived. Since elevated CO_2_ stimulated growth and counteracted stress [45], the appearance of trehalose in microgravity-grown plants (Figure 4) corresponds with the notion that trehalose enhances stress resistance in radish [46,47]. Therefore, trehalose may be a threshold indicator of stress experienced in space, given that trehalose was detected in less than 1/3 of the space-grown plants.

Similarly, maltose, typically the product of starch hydrolysis by β-amylase, appeared in plants grown at KSC and ISS and could therefore be a response to elevated CO_2_. However, the distribution of starch in radish bulbs is not universal but can be found in regular, Earth-grown plants (Figure S3). Therefore, the presence of maltose may be indicative of starch as much as the maltose content.

*Gravity effects:* Previous research on space-grown plants reported a 75% increase in 3-butenylglucosinolate in stems of *Brassica rapa*, a close relative to radish [48]. Our data were limited to the typically consumed bulb material and did not include leaf material. No gravity-related effects on GSL levels or composition were detected. This observation indicates that gravity is not a relevant factor for GSL metabolism in radish bulbs and suggests that radish is a suitable ‘space crop’ in that it retains its nutritional value with respect to not only the GSLs but also the sugars, even under altered gravity or atmospheric CO_2_ conditions. Carbon accumulation depends on the nitrogen source and concentration [49], but that aspect of secondary metabolism was not investigated. However, the fertilizer load in the space and ground experiments was deemed adequate for at least two grow-outs [21]. Because each experiment used only the original quantity of nutrients (half-strength modified MS medium, [21]), changes in GSL or sugars because of a nitrogen imbalance or other nutrient-related effects can be ruled out.

This is the first report that evaluates the metabolic range of GSLs and sugars in space-grown plants. The variability of GSLs and carbohydrate quantities suggests that plant metabolism is adaptable to changing gravity and atmospheric CO_2_ conditions. It also helps alleviate concerns that space cultivation and reported associated stress responses [50,51,52,53] do not represent a decisive factor for plant productivity under micro- or reduced (i.e., lunar) gravity. However, secondary metabolism should be regularly examined in space-grown plants as it is a sensitive indicator of growth conditions and to detrimental effects of space cultivation, such as volatile organic compounds (VOCs), reduced mixing of the gas phase, or water distribution.

## 4. Materials and Methods

### 4.1. Plant Cultivation

**Figure 6:**
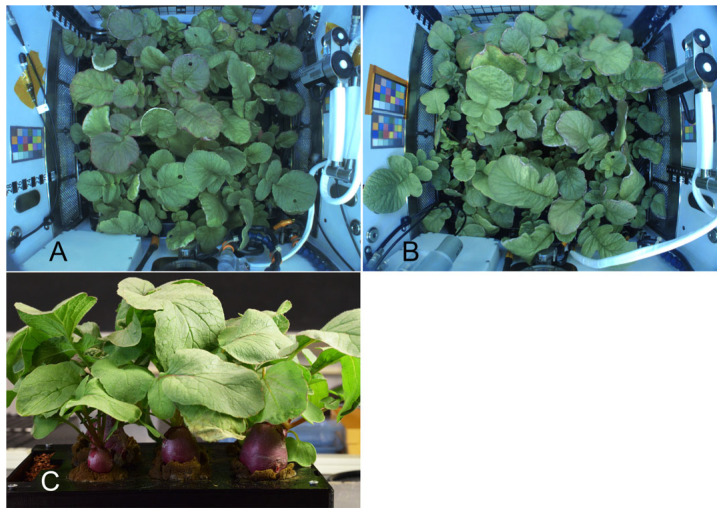
Radish plants grown in the ISS (**A**), the Kennedy Space Center (**B**), and the ULL laboratory (**C**) for 27 days before harvesting. The average bulb mass per experiment (16 ± 7 g) was similar for all three conditions [21].

The radish studied was *Raphanus sativus* L. variety “Cherry Belle”. Seeds were sanitized and grown in arcillite that was fertilized in half-strength MS medium and grown as described previously [21]. The space experiment was launched on 2 October 2020, and two grow-outs were completed in November and December 2020 in the APH with a CO_2_ concentration of ~2500 ppm (Figure 6A). Ground controls were assessed in December 2020 at the Kennedy Space Center (KSC, ~2500 ppm CO_2_, Figure 6B) and in May 2021 at the University of Louisiana Lafayette (ULL) laboratory (~400 ppm CO_2_, Figure 6C). Immediately after harvest, the tissues were frozen (–80 °C). The ISS samples were returned in July 2021. The bulb material of all samples was cut into 5–7 mm cubes and lyophilized before further processing.

### 4.2. Glucosinolate Analysis

The extraction of GSLs from radish bulbs was carried out according to the method described by Maldini et al. [54], with the following modifications: The freeze-dried cut bulb pieces were individually ground to fine powders using the TissueLyser II (Qiagen, Germantown, United States) for 30 s at a frequency of 30 Hz. Then, 15–25 mg of each sample was transferred to a 2 mL screw cap microcentrifuge tube, to which a solution of sinigrin (0.1 mM) in methanol–water (1:1, *v*/*v*) was added in the amount of 1 mL per 50 mg of tissue powder. Each suspension was incubated at 70 °C for 30 min. After cooling, each tube was centrifuged at 16,000× *g* (4 °C, 15 min), with the corresponding supernatant collected, diluted 1 to 10 (1 part supernatant + 9 parts methanol–water (1:1, *v*/*v*)), and subjected to LC-MS analysis.

### 4.3. UPLC-qTOF-MS Analysis

GSL analyses were performed as described in Glauser et al. [23], with the following modifications: A Synapt G2-S quadrupole time-of-flight mass spectrometer connected to an Acquity UPLC system with an Acquity photodiode array detector (all Waters, Milford, MA, USA) was used for LC-MS analysis. Analytes were separated on an Acquity UPLC CSH C18 column (100 × 2.1 mm, particle size 1.7 µm, Waters) using a linear gradient. The mobile phase was composed of water (solvent A) and acetonitrile (solvent B), each supplemented with 0.05% formic acid. The flow rate was 400 µL min^−1^. The gradient started at 2% B and reached 45% B at 6 min, increased to 99% B at 6.5 min, and held for 3 min, then returned to 2% B within 1 min and equilibrated for 3.5 min. Samples were injected as 2 μL volumes. The column temperature was 25 °C. Mass spectra were collected in the negative electrospray ionization mode, and data were acquired in both centroid and resolution modes over a range of 50–1000 *m/z* with a scan time of 0.3 s. The capillary charge was 2.5 kV, and the sampling cone was at 40 V. The source temperature was 100 °C, the desolvation temperature was 150 °C, and cone gas and desolvation gas flow were set at 0 and 600 L h^−1^, respectively. Fragmentation was carried out via recording exact-mass precursor and fragment ions (MS^E^ mode) using a collision energy ramp of 20–60 eV. The mass spectrometer was calibrated using sodium formate with a 95% confidence cutoff of 1 ppm. Leucine enkephalin was infused continuously during the runs and used for real-time mass correction. Data analysis was performed using MassLynx 4.1, and metabolomics data processing was conducted using the built-in TargetLynx module (Waters), where the *m/z* window was set to 15 ppm of the observed mass for all targets. RAW data files were converted to mzML files using ProteoWizard’s MSConvertGUI (64-bit) software. mzML files were then processed in RStudio (version 2024.04.0 Build 735) using the XCMS and CAMERA packages with the following parameters: prefilter = c(7,2000), snthr = 20, ppm = 1.5, peak width = c(10,60), integrate = 1, and mzdiff = 0.005 [1].

The TSV and CSV files generated after XCMS and CAMERA processing were combined and further analyzed manually. Data were normalized to the internal standard sinigrin [Rt 2.83 min, *m/z* 358.0267 (calculated for sinigrin)] for statistical analysis (Table 1).

The above chromatographic conditions did not separate 4-hydroxglucobrassicin from an unidentified compound of [M–H]^–^ 479.0397. Both components were subsequently completely separated using an Acquity BEH C18 column and eluted, as shown in Table 1. The fragmentation of [M–H]^–^ 479.0397 also did not result in any known GSL fragments.

### 4.4. Sugar Quantification

Sugars were extracted from 40 to 90 mg of lyophilized tissue that was weighed and transferred to 15 mL Falcon tubes. Then, 10 mL of nanopure water was added, and the suspension was autoclaved for 30 min [55]. An aliquot (2–2.5 mL) was filtered through a 13 mm, 0.45 μm nylon syringe filter (Biomed Scientific), and 200 μL of each eluant was mixed with 800 μL acetonitrile. For each sample, 20 μL was applied to a Hypersil-gold amino column, 250 × 4.6 mm (25,705–25,4630, Thermo Fisher Scientific, Waltham, MA, USA), and isocratically eluted with 80% acetonitrile/20% water (*v*/*v*) at 35 °C with a flow rate of 1.2 mL min^–1^ on a Shimadzu Prominence HPLC LC-20AD system with an evaporative light-scattering detector ELSD-LTII with N_2_ @150 kPa (Peak ABN2ZA, Inchinnan, Scotland, UK). Quantification was based on calibration curves from injections between 0 and 16 μg of individual saccharides, with identities based on coincident retention times (Table 2). Quantities below 0.5 μg per sample were not included in the resulting data sets.

## 5. Conclusions

Understanding plant growth in confined environments, especially under altered gravity and light conditions, poses significant challenges that are typically addressed using genetic tools such as RNA-seq and qPCR. In contrast, metabolomic studies remain relatively rare, although they highlight changes in secondary metabolite profiles. Our study is the first to assess changes in GSLs—a valuable family of sulfur-containing phytochemicals—and simple carbohydrates in space-grown plants. We found that variations in both compound classes were more strongly influenced by elevated CO_2_ levels than by microgravity. These findings demonstrate the capacity of plants to adapt to changes in gravity and atmospheric composition and offer an optimistic outlook for future plant cultivation on the Moon or Mars.

## Figures and Tables

**Table 1:** Retention times (Rt) and calculated HRMS molecular ions (M–H)^–^, fragmentation patterns, and identity of analyzed major glucosinolates.

Annotation	Rt, min	Observed Mass ^‡^ [M–H]^–^	Calculated Mass [M–H]^–^	Error, ppm	Ion Fragments **	Molecular Formulae
Glucoraphenin	2.53	434.0248	434.0249	0.23	259, 97, 96	C_12_H_20_NO_10_S_3_
4-Hydroxyglucobrassicin ^§^	4.12	463.0500	463.0481	4.10	259, 97, 96	C_16_H_19_N_2_O_10_S_2_
Glucoerucin	4.59	420.0452	420.0457	1.19	259, 97, 96	C_12_H_22_NO_9_S_3_
Glucoraphasatin *	4.60	418.0298	418.0300	0.48	259, 97, 96	C_12_H_20_NO_9_S_3_
Glucobrassicin	4.80	447.0529	447.0532	0.67	259, 97, 96	C_16_H_19_N_2_O_9_S_2_
4-Methoxyglucobrassicin	5.19	477.0645	477.0638	1.47	259, 97, 96	C_17_H_21_N_2_O_10_S_2_
3-Methylpentyl GSL	5.50	402.0881	402.0861	4.97	259, 97, 96	C_13_H_24_NO_9_S_2_
4-Methylpentyl GSL	5.62	402.0877	402.0861	3.98	259, 97, 96	C_13_H_24_NO_9_S_2_
*n*-Hexyl GSL	5.75	402.0877	402.0861	3.98	259, 97, 96	C_13_H_24_NO_9_S_2_

^‡^ Observed masses ([M–H]^–^) are given as mean values for all analyses. ^§^ 4-Hydroxyglucobrassicin co-eluted with another unidentified compound at 4.12 min with [M–H]^–^ of 479.0397 using an Acquity UPLC CSH C18 column. The same analyses using an Acquity UPLC BEH C18 column instead separated 4-hydroxyglucobrassicin from the unidentified compound, but the unidentified compound ([M–H]^–^ 479.0397) did not result in any known GSL fragments. * Confirmed using glucoraphasatin as an authentic standard. ** Fragments *m/z* 259, 97, and 96 are representative of sulfated glucose (*m/z* 259), HSO_4_^2−^ (*m/z* 97), and SO_4_^2−^ (*m/z* 96), respectively, and these are major universal GSL fragment ions [25,29,30].

**Table 2:** Radish bulb carbohydrates and their HPLC-separated retention times relative to authentic standards as described in Materials and Methods.

Carbohydrate	Rt, min
Rhamnose	4.54
Fucose	5.08
Psicose	5.09
Xylose	5.11
Arabinose	5.55
Fructose	6.05
Mannose	6.67
Sorbitol	6.88
Mannitol	7.03
Glucose	7.05
Galactose	7.35
Galactosamine	9.21
Sucrose	10.19
Myo-inositol	11.35
Maltose	12.17
Lactose	13.93
Trehalose	14.31

## Data Availability

The data presented in this study are available upon request from the corresponding author.

## Supplementary Materials

The following supporting information can be downloaded at: https://www.mdpi.com/article/10.3390/plants14132063/s1, Figure S1: Representative examples of glucosinolates in radish bulb (KSC ground grown control). A, Total ion chromatogram (TIC) of glucosinolates **1**–**9**; Selected ion monitoring (SIM) of glucoraphenin **1** (B), 4-hydroxyglucobrassicin **2** (C); glucoerucin **3** (D), glucoraphasatin **4** (E); glucobrassicin **5** (F); 4-methoxyglucobrassicin **6** (G); 3-methylpentyl GSL, **7**, 4-methylpentyl GSL **8**, and *n*-hexyl GSL **9** (H); sinigrin (internal standard) (I); Figure S2: Chemical structures of radish glucosinolates and internal standard sinigrin; Figure S3: Sections through radish (*Raphanus sativus*, var Cherry Belle) bulb tissue from a specimen without starch (A) and a different bulb with many Lugol-stained starch grains (B). Bar: 25 µm.

## Abbreviations

The following abbreviations are used in this manuscript:

APH	Advanced Plant Habitat
GSL	Glucosinolate
KSC	Kennedy Space Center
ISS	International Space Station
LC-MS	Liquid chromatography–mass spectrometry
UPLC-qTOF-MS	Ultra-high performance liquid chromatography with quadrupole time-of-flight mass spectrometry
