# Supplementary material for: Glucosinolate and Sugar Profiles in Space-Grown Radish
Source: Plants (Basel). 2025 Jul 6;14(13):2063. doi: 10.3390/plants14132063 (PMC12252257; doi:10.3390/plants14132063)
Supplement: Supplementary file 1 [file plants-14-02063-s001.zip › plants-3553853-supplementary.pdf]

plants

MDPI

Supplementary Materials: The following supporting information can be downloaded at: www.mdpi.com/xxx/s1, Figures S1, S2, S3.

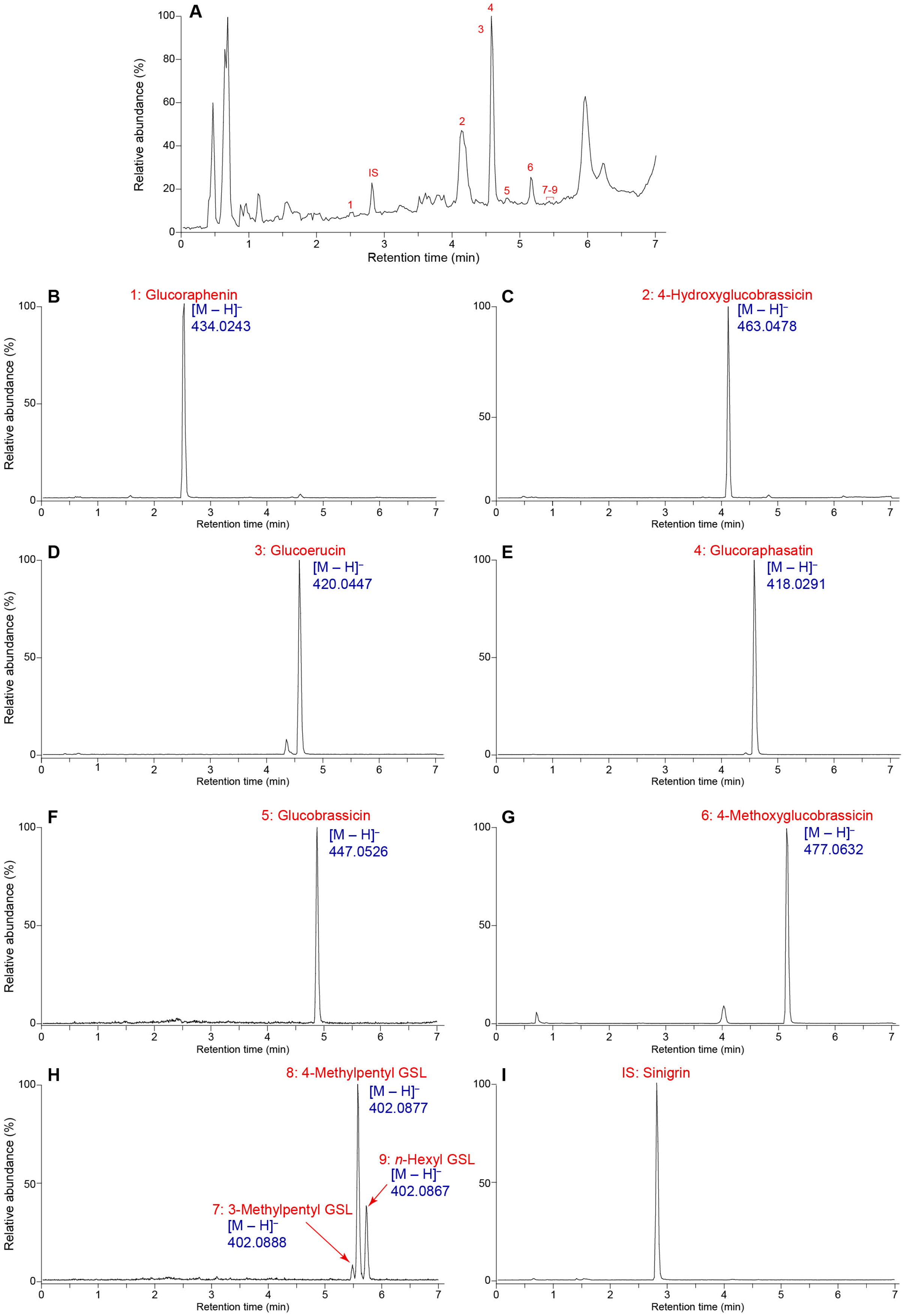

Figure S1: Representative examples of glucosinolates in radish bulb (KSC ground grown control). **A**, Total ion chromatogram (TIC) of glucosinolates **1** - **9**; Selected ion monitoring (SIM) of glucoraphenin **1** (**B**), 4-hydroxyglucobrassicin **2** (**C**); glucoerucin **3** (**D**), glucoraphasatin **4** (**E**); glucobrassicin **5** (**F**); 4-methoxyglucobrassicin **6** (**G**); 3-methylpentyl GSL, **7,** 4-methylpentyl GSL **8,** and *n*-hexyl GSL **9** (**H**); sinigrin (internal standard) (**I**).

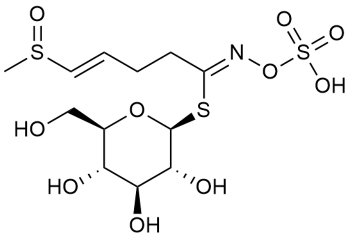

1: Glucoraphenin

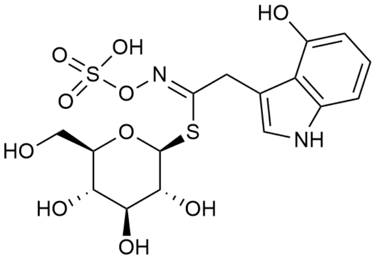

2: 4-Hydroxyglucobrassicin

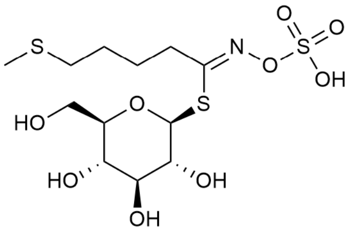

3: Glucoerucin

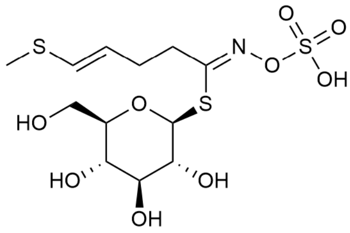

4: Glucoraphasatin

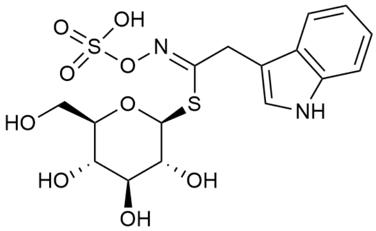

5: Glucobrassicin

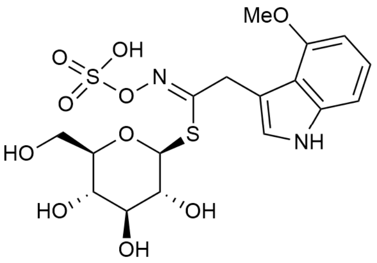

6: 4-Methoxyglucobrassicin

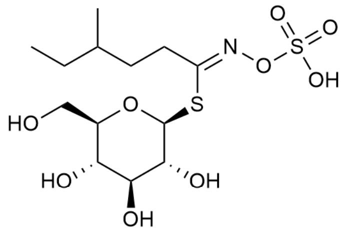

7: 3-Methylpentyl glucosinolate

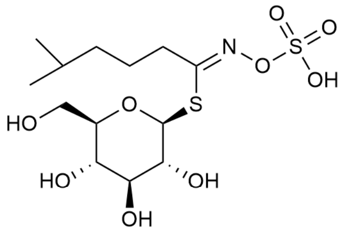

8: 4-Methylpentyl glucosinolate

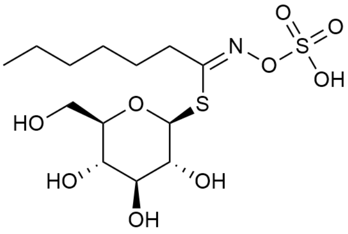

9: *n*-Hexyl glucosinolate

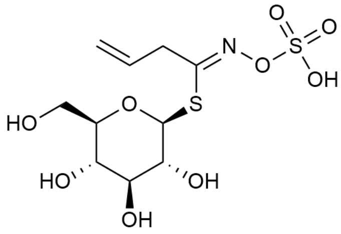

IS: Sinigrin

Figure S2: Chemical structures of radish glucosinolates and internal standard sinigrin.

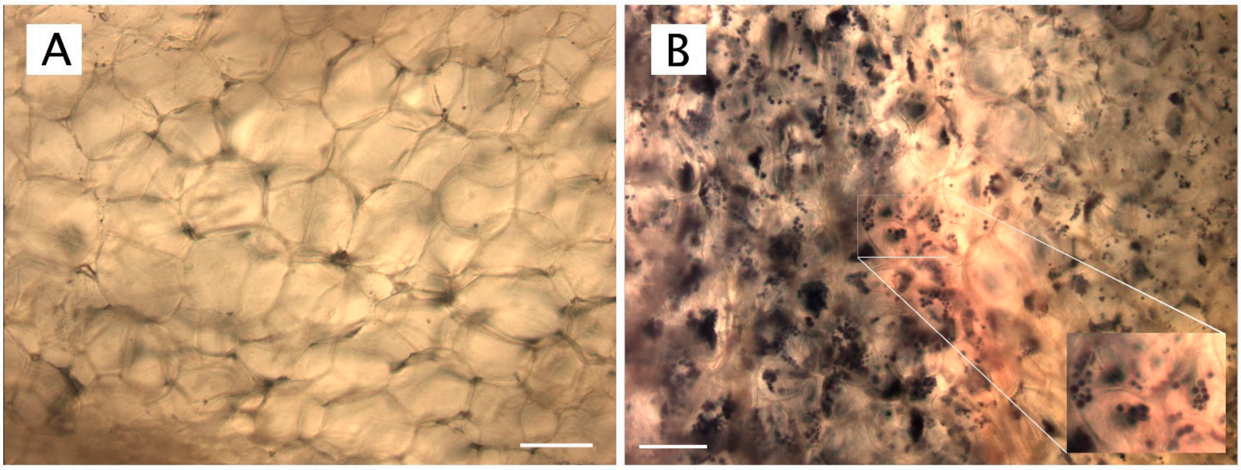

Figure S3. Sections through radish (*Raphanus sativus*, var Cherry Belle) bulb tissue from a specimen without starch (**A**) and a different bulb with many Lugol-stained starch grains (**B**). Bar: 25 μm.
